# Deletion of 7q33-q35 in a Patient with Intellectual Disability and Dysmorphic Features: Further Characterization of 7q Interstitial Deletion Syndrome

**DOI:** 10.1155/2015/131852

**Published:** 2015-05-03

**Authors:** Kristen Dilzell, Diana Darcy, John Sum, Robert Wallerstein

**Affiliations:** ^1^Department of Medical Genetics, University of Pennsylvania, Philadelphia, PA 19104, USA; ^2^Silicon Valley Genetics Center, Santa Clara Valley Medical Center, San Jose, CA 95128, USA; ^3^Pediatric Neurology, Santa Clara Valley Medical Center, San Jose, CA 95128, USA

## Abstract

This case report concerns a 16-year-old girl with a 9.92 Mb, heterozygous interstitial chromosome deletion at 7q33-q35, identified using array comparative genomic hybridization. The patient has dysmorphic facial features, intellectual disability, recurrent infections, self-injurious behavior, obesity, and recent onset of hemihypertrophy. This patient has overlapping features with previously reported individuals who have similar deletions spanning the 7q32-q36 region. It has been difficult to describe an interstitial 7q deletion syndrome due to variations in the sizes and regions in the few patients reported in the literature. This case contributes to the further characterization of an interstitial distal 7q deletion syndrome.

## 1. Introduction

While a syndrome due to a terminal deletion of 7q has been described [1, 2], interstitial deletions in 7q have been less well categorized. Previously reported 7q interstitial deletions have spanned different chromosomal segments of varying sizes, making it difficult to define a 7q interstitial deletion syndrome. Furthermore, many of these patients were reported prior to the implementation of genome-wide array comparative genomic hybridization (array CGH) and knowledge of many gene locations and functions.

We report on a patient with a 9.92 Mb interstitial chromosome deletion of 7q33-q35 and summarize the clinical features seen in this patient, other patients with 7q33-q35 deletions, and patients with other 7q interstitial and terminal deletions, to help understand the range of phenotypes seen in patients with interstitial 7q deletions. We also review the known genes in this region to assess relevant genotype-phenotype correlations.

## 2. Case Presentation

The patient, now 16 years old, was born to a 23-year-old G4, P3→4 mother and 30-year-old father. During the pregnancy there was concern for decreased fetal movement. Labor was induced due to fetal distress at 36 weeks' gestation. Birth weight was 2614 g (<3%) and birth length was 44.5 cm (<3%). She had a positive toxicology screen for barbiturates and was noted to have large open fontanels. The patient remained in the hospital for 5 days after birth due to feeding difficulties.

The patient came to attention again around 6 months of age due to developmental delay and was evaluated by neurology. At around one year of age, the patient also began having seizure activity. EEG results were normal; a brain MRI at 14 months of age showed prominent CSF space posterior to cerebellar vermis, most likely representing large cisterna magna. A karyotype revealed abnormal results, 46,XX,del (7)(q32-q34). The patient's mother had a normal karyotype and her father remains unavailable for testing but is not known to have any features similar to the patient; her deletion is presumed to be de novo based on its large size. Her seizures resolved by 2.5 years of age.

The patient has had multiple recurrent infections throughout her life; by 28 months of age, she had been hospitalized for pneumonia 3 times. The patient has continued to experience multiple upper respiratory infections, recurrent otitis media, and urinary tract infections. Immunology work-up was normal; therefore the patient is not suspected of having a primary immunodeficiency. The patient has a history of chronic otitis media and abnormal tympanometry and audiology examinations; she had ear tubes placed at 30 months of age. An audiogram performed at age 8 showed right moderate conductive hearing loss and left borderline to mild hearing loss, and hearing aids were recommended. The patient was first evaluated by ophthalmology at 33 months and noted to have hyperopia and bilateral astigmatism but no evidence of optic atrophy, as reported in some patients with 7q interstitial deletions.

At age 7 the patient was diagnosed with likely obstructive sleep apnea syndrome. She has a history of snoring and insomnia. In the same year the patient was noted to have elevated ALT and AST levels, which have progressively increased; suspected etiology is nonalcoholic steatohepatitis. A liver biopsy revealed macrovesicular steatosis and a modest number of lymphocytes in a few portal triads. The patient has also been followed up by endocrinology since age 10 due to abnormal weight gain, acanthosis nigricans, and insulin resistance. She was diagnosed with type II diabetes at age 11 and started on metformin. Additionally, the patient is reported to have large, foul smelling stools since 12 years of age.

At age 14, the patient developed hemihypertrophy on her right side and swelling of the right side of her face in the morning that regresses during the day. The hypertrophy interferes with walking and causes pain in her right foot. Genetic testing for Beckwith-Wiedemann syndrome was negative. A CT scan of the lower extremities showed increased diameter of the right leg in relation to the left, with a relative increase in both subcutaneous fat and muscle size. The increased musculature may represent compensatory hypertrophy secondary to the increased overall mass of the right lower extremity, but idiopathic or genetic reasons for hemihypertrophy cannot be excluded. There is no visible mass to account for the apparent disparity in size. An abdominal ultrasound showed a diffusely echogenic liver, compatible with fatty infiltration. Previous echocardiograms and EKGs have been normal. At age 15, the patient's lower left leg also appeared to be growing in size. The patient currently has menstrual cycles two times per month. She developed a hyperpigmented spot on her forehead at age 15.

The patient has a history of significant developmental delay and intellectual disability. She sat at one year and walked at two years. At 28 months she had one or two understandable words but was mostly nonverbal. At age 7 her vocabulary was 15 words, but she did not yet put words together in phrases or sentences. At age 13 she was in a special education class in the 7th grade, assessed to be at a 3-year-old level. Currently at age 16, she is not able to name colors or count, but she can write her name, speak in simple sentences, and name some body parts. She has not lost any skills over time. The patient has a history of violent behaviors towards others and self-injurious behavior, including skin picking which began around 3 years of age and continues to present. Methylation analysis for Prader-Willi syndrome (due to features of obesity, intellectual disability, and skin-picking behaviors) and testing for cystic fibrosis (due to bowel concerns) were both normal.

The patient has dysmorphic features including small ears, a right preauricular ear pit, a large mouth with downturned corners, a smooth philtrum, a thin upper lip, hypertelorism, bulbous nose, a low posterior hairline, and a short neck. Lumbar lordosis was first noted around 3 years of age and continues to present. The patient also has a small umbilical hernia.

The patient's family history is not significant for chromosome abnormalities, developmental delay, or intellectual disability. She has six maternal half-siblings who all reportedly have ADD and several paternal half-siblings who have no known medical or developmental concerns and are not available for testing. She has no full siblings. She is of African American and Northern European descent.

SNP microarray was performed by Integrated Genetics using the Affymetrix Cytoscan HD platform, using 743,000 SNP probes and 1,953,000 NPCN probes. A 9.22 Mb interstitial deletion of 7q33-7q35 was identified, arr 7q33-q35 (133,297,307–143,218,955) × 1.

## 3. Discussion

Table 1 reports on the clinical features of other patients with 7q interstitial deletions overlapping the same region as our patient. Malmgren et al. reported on a family with an interchromosomal insertion of 7q33-q34 into 6p25, resulting in some family members who were balanced carriers giving rise to three offspring with a 7q33-q34 deletion of 7.6 Mb [3]. All three individuals with the deletion are reported to have intellectual disability, a long philtrum, a thin upper lip, a bulbous nose, a large mouth, and hypertelorism, as does our patient. Two of those three patients described by Malmgren had ear abnormalities and recurrent infections, and two patients had growth retardation or short stature. As shown in Table 1, these features have also been reported in other patients with interstitial deletions of varying sizes between 7q31-7q35. Our patient's facial appearance is quite similar to the patients described by Malmgren, particularly individuals IV: 6 and III: 3.

In addition to the features described in previous patients, our patient presents with additional findings of sleep apnea and insomnia, type 2 diabetes, obesity, echogenic liver, frequent menstruation, aggressive and self-mutilating behaviors, and her most recent concern of hemihypertrophy. Currently it is unclear which of these features can be explained by the patient's deletion. However, Rossi et al. describe a patient with a 7q33-q35 deletion who also presents with obesity and sleep disturbances, as well as mood shifts [4].

The patients currently reported with interstitial 7q deletions show some phenotypic differences from patients with 7q34-ter or 7q35-ter deletions. Holoprosencephaly and Currarino triad have been reported in terminal deletions [5]; these conditions are attributed to the loss of* MNX1* and* SHH*. Additional features reported in 7q terminal deletions include microcephaly, cleft lip and palate, large ears, abnormal genitalia, and cardiac anomalies [6]. However, patients with both interstitial and terminal deletions in this region have had intellectual disability, similar dysmorphic facial features, and growth abnormalities [6, 7].

Rush et al. recently reported on a patient with a 7q34-q36.1 interstitial deletion and reviewed patients with similar deletions in overlapping q34-q36 regions [7]. Although this is a slightly different location than our patient's, these patients also show some phenotypic overlap with our case and others with q32-q35 deletions, including facial characteristics such as bulbous nasal tip and broad nasal root, intellectual disability, impulsive behavior, hearing loss, and seizures. The shared phenotypic characteristic of these bordering deleted segments may help further narrow down the 7q34-q35 region as the responsible area for these shared features.

There are 64 OMIM-described genes known to be located in the particular area of 7q deleted in our patient (Table 2). None of these genes are known to be imprinted. Like the patients previously described with similar deletions, our patient's deleted region includes the genes* CNOT4*,* MTPN*,* CHRM2*, and* PTN*, which are of special interest given their involvement in nervous system development [3, 8]. Deletions of these genes could cause differences in nervous system development that could explain the intellectual disability and behavioral concerns seen in our patient and individuals with similar 7q deletions. Several deleted genes have also been implicated in autosomal dominant movement disorders including* CLCN1* (Thomson dominant myotonia) and* DHMN1* (distal hereditary motor neuronopathy). While our patient does not carry a label of either of these diagnoses, she does have increasing difficulty with walking and movement.

The gene* TBXAS1*, which encodes thromboxane synthase involved in platelet aggregation and clotting, may be of interest given that our patient has two menstrual cycles a month, and two other patients with deletions spanning the 7q33 to 7q36 region have had the opposite phenotype of primary amenorrhea [4, 9]. However, while homozygous and compound heterozygous mutations are known to cause Ghosal hematodiaphyseal syndrome, the significance of haploinsufficiency is unclear. The patient also has several deleted genes that encode the T-cell receptor beta chain, and while interesting given her immunological concerns, no phenotype has been reported with alterations to these genes to date.

Mutations in* BRAF* have been associated with RASopathies, most commonly cardiofaciocutaneous syndrome (CFC) but also Noonan and LEOPARD syndrome. Mutations causing these syndromes lead to activation of the RAS-MAPK pathway.* BRAF* haploinsufficiency is not expected to be a causative mechanism of CFC; however it is interesting to note that our patient's course facial features overlap with many course facial features of CFC, including bulbous nasal tip and depressed nasal bridge, hypertelorism, downslanting palpebral fissures, and prominent philtrum.

*OTCS2* has been implicated in otosclerosis, resulting in conductive hearing loss, which is of interest given our patient's history of conductive hearing loss and hearing loss seen in other patients with similar deletions. Other genes in the deleted region have shown associations with other diseases not currently seen in our patient, including* HIPK2* with leukemia,* ATP6V0A4* with renal tubular acidosis,* TRIM24* with papillary thyroid carcinoma,* PRSS1* with pancreatitis, and* GPDS1* with glaucoma and optic nerve degeneration.

## 4. Conclusion

The patient presented exhibits similar features to other individuals with similar deletions, particularly intellectual disability and similar dysmorphic facial features, further helping to characterize a 7q interstitial deletion syndrome encompassing the q33-q35 region. This patient also exhibits unique features including recent onset of hemihypertrophy, frequent menses, and self-mutilation behaviors. There are many known genes in this region that play important roles in brain development and organ function whose haploinsufficiency could contribute to the features seen in our patient and others with similar deletions.

## Figures and Tables

**Table 1:** Clinical features of individuals with interstitial 7q33-q35 deletions.

Patient demographics	Present case	Malmgren et al. [3]			Nielsen et al. [10]			Stallard and Juberg [2]	Verma et al. [11]	Petrin et al. [8]	Rossi et al. [4]	Fagan et al. [12]
Patient		IV: 6	III: 1	III: 3	III: 11	III: 10	III: 8					
Deletion region	q33-q34	q33-q34	q33-q34	q33-q34	q32-q34	q32-q34	q32-q34	q31-q34	q33-q35	q33-q35	q33-q35	q35
Gender/age	F/15	M/15	M/40	F/25	F/6	M/6	F/13.5	F/1	F/4	M/22	F/adult	F/11
Neurological/development												
Intellectual disability	+	+	+	+	+	+	+	NA	+	−	+	+
Developmental delay	+	+	+	+	+	+		+	+	+	+	+
Speech delay/disorder	+	+						NA	+	+	+	+
Abnormal brain MRI	+	+								+		
Mood shifts	+										+	
Self-mutilating behaviors	+											
Seizures	+						+		+		+	
Sleep difficulty	+										+	
Diparesis/truncal ataxia											+	
Craniofacial												
Microcephaly								+	+			
Hypertelorism	+	+	+	+	+	+	+				+	
Epicanthal folds		+						+				+
Broad/depressed nasal bridge		+			+	+	+			+	+	
Bulbous nose	+	+	+	+	+	+	+	+			+	+
Large mouth	+	+	+	+	+	+	+				+	
Long philtrum	+	+	+	+	+	+					+	
Thin upper lip	+	+	+	+	+	+		+				
Micrognathia		+										+
Low set ears/ear		+	+		+	+		+				+
Preauricular ear pits	+										+	
Growth												
Slow growth/short stature		−	+	+	−	+	+	+	−			
Truncal obesity	+										+	
Hemihypertrophy	+											
Hand and feet differences		+								+	+	
Other												
Ophthalmologic abnormality	+						+		−			+
Hearing deficit	+	+							+	−		
Repeated infections	+	+	+		+			+	+			
Primary amenorrhea	−	N/A	N/A			N/A				N/A	+	
Frequent menses	+	N/A	N/A			N/A				N/A	−	
Umbilical hernia	+	+										

**Table 2:** Known deleted genes in 7q33-q35 region in OMIM.

Known deleted genes in OMIM		
AGK	HIPK2	TAS2R3
AKR1B1	KEL	TAS2R4
AKR1B10	KIAA1549	TAS2R5
AKR1D1	LUC7L2	TAS2R38
ATP6V0A4	LUZP6	TAS2R40
BPGM	MGAM	TAS2R41
BRAF	MTPN	TAS2R60
CALD1	MRPS33	TBXAS1
CASP2	NUP205	TRIM24
CHRM2	OTSC2	TRBC1
CLCN1	PARP12	TRBC2
CLEC5A	PIP	TRBD1
CNOT4	PTN	TRBD2
CREB3L2	PRSS1	TRBJ@
D7S437	PRSS2	TRBV@
DFNB13	SLC13A4	TRPV5
DGKI	SLC35B4	TRPV6
DHMN1	SLI4	UBN2
EPHA1	SSBP1	WEE2
EPHB6	STRA8	ZC3HAV1
GPDS1	SVOPL	ZYX
GSTK1		
